# Profound Effects of *Aggregatibacter actinomycetemcomitans* Leukotoxin Mutation on Adherence Properties Are Clarified in *in vitro* Experiments

**DOI:** 10.1371/journal.pone.0151361

**Published:** 2016-03-15

**Authors:** Senthil Kumar Velusamy, Vandana Sampathkumar, Dipti Godboley, Daniel H. Fine

**Affiliations:** Department of Oral Biology, Rutgers School of Dental Medicine, 185 South Orange Ave, Newark, New Jersey, United States of America; LSU Health Sciences Center School of Dentistry, UNITED STATES

## Abstract

Leukotoxin (Ltx) is a prominent virulence factor produced by *Aggregatibacter actinomycetemcomitans*, an oral microorganism highly associated with aggressive periodontitis. Ltx compromises host responsiveness by altering the viability of neutrophils, lymphocytes, and macrophages. Previously, we developed a Rhesus (Rh) monkey colonization model designed to determine the effect of virulence gene mutations on colonization of *A*. *actinomycetemcomitans*. Unexpectedly, an *A*. *actinomycetemcomitans* leukotoxin (*ltx*A) mutant (RhAa-VS2) failed to colonize in the Rh model. No previous literature suggested that Ltx was associated with *A*. *actinomycetemcomitans* binding to tooth surfaces. These results led us to explore the broad effects of the *ltx*A mutation *in vitro*. Results indicated that LtxA activity was completely abolished in RhAa-VS2 strain, while complementation significantly (*P*<0.0001) restored leukotoxicity compared to RhAa-VS2 strain. RT-PCR analysis of *ltx* gene expression ruled out polar effects. Furthermore, binding of RhAa-VS2 to salivary-coated hydroxyapatite (SHA) was significantly decreased (*P*<0.0001) compared to wild type RhAa3 strain. Real time RT-PCR analysis of the genes related to SHA binding in RhAa-VS2 showed that genes related to binding were downregulated [*rcp*A (*P* = 0.018), *rcp*B (*P* = 0.02), *tad*A (*P* = 0.002)] as compared to wild type RhAa3. RhAa-VS2 also exhibited decreased biofilm depth (*P* = 0.008) and exo-polysaccharide production (*P*<0.0001). Buccal epithelial cell (BEC) binding of RhAa-VS2 was unaffected. Complementation with *ltx*A restored binding to SHA (*P*<0.002) but had no effect on biofilm formation when compared to RhAa3. In conclusion, mutation of *ltx*A diminished hard tissue binding *in vitro*, which helps explain the previous *in vivo* failure of a *ltx*A knockout to colonize the Rh oral cavity. These results suggest that; 1) one specific gene knockout (in this case *ltx*A*)* could affect other seemingly unrelated genes (such as *rcp*A, *rcp*B *tad*A *etc)*, and 2) some caution should be used when interpreting the effect attributed to targeted gene mutations when seen in a competitive *in vivo* environment.

## Introduction

*Aggregatibacter actinomycetemcomitans* is a Gram negative coccobacillus associated with localized aggressive periodontitis (LAP), a severe form of periodontal disease [1]. Moreover, a specific clone of this organism, the JP2 clone, has been described as a unique, highly destructive pathogenic form of this bacteria primarily because it is known to produce elevated levels of a host cell-killing toxin, leukotoxin [2]. While non-JP2 clones are more prevalent world-wide and are also associated with aggressive periodontal disease, the JP2 leukotoxin producing clone is rarely found in healthy subjects and is almost always associated with a more rapidly progressive and advanced stage of disease[3]. The key distinction between the “commensal form” of *A*. *actinomycetemcomitans* and its “pathogenic form” (the JP2 strain) is thought to be related to its increased level of leukotoxin production [4]. Ltx has been shown to cause death of human polymorphonuclear leukocytes (PMNs), monocytes, and lymphocytes, and consequently Ltx is assumed to protect *A*. *actinomycetemcomitans* against surveillance and destruction by host cells [5]. In a mixture of low-leukotoxin producing bacteria, human serum and PMNs, the bacteria are efficiently phagocytized and killed at a ratio of 25 bacteria/PMN i*n vitro* [6]. In contrast, in the presence of high-leukotoxin producing bacteria under the same physiological conditions, the PMNs fail to phagocytize and kill the bacteria (6). On a clinical level, individuals who carry the high Ltx producing strains show a substantially increased risk for periodontal attachment loss as compared to those individuals with the low Ltx producing strains [2].

Our group has focused on *A*. *actinomycetemcomitans* survival in the face of microbial/microbial and host/microbial interactions [3, 7–9]. Recently, we developed a Rhesus (Rh) monkey model designed to study the colonization and persistence of *A*. *actinomycetemcomitans* strains inoculated into the mouths of Rh monkeys [10]. We chose Rh monkeys because they typically harbor *A*. *actinomycetemcomitans* and have an oral flora and anatomy that resembles that found in humans[10]. Our goal was to see how various key *A*. *actinomycetemcomitans* virulence factors affect colonization and survival in a competitive oral environment. Initial studies compared colonization of *A*. *actinomycetemcomitans* strains derived from humans (Hu) as compared to those derived from Rh monkeys. In spite of repeated inoculation human *A*. *actinomycetemcomitans* (HuAa), could not be recovered at any sampling time over a 4-week period. On the other hand, *A*. *actinomycetemcomitans* was successfully recovered at all time points in all animals inoculated with a strain derived from a Rh monkey [10]. In a subsequent study it was shown that a wild type (RhAa3) strain and a quorum sensing deficient *A*. *actinomycetemcomitans* strain (LuxS mutant) could colonize but a *ltx*A knockout *A*. *actinomycetemcomitans* strain (RhAa-VS2) could not colonize (Unpublished data). This unforeseen failure of the *ltxA* mutant *A*. *actinomycetemcomitans* strain (RhAa-VS2) to colonize any area in the mouths of Rh monkeys provoked us to do *in vitro* quantitative assessment of biofilm formation and soft and hard tissue binding in a *ltx*A knock-out in comparison to its wild-type and complemented strains.

Overall, *A*. *actinomycetemcomitans* has been isolated from the oral cavity of humans [11, 12] and non-human primates [10, 13] and belongs to the Haemophilus, Actinobacillus, Cardiobacteria, Eikenella, Kingella (HACEK) group of organisms. It has also been associated with non-oral systemic infections [13–15]. *A*. *actinomycetemcomitans* is highly adaptable and possesses a variety of virulence genes that produce toxins, adhesins, invasins, and antibiotic resistance factors [16]. The products of these virulence genes provide *A*. *actinomycetemcomitans* with the essential properties that enable it to colonize and survive in the highly variable and competitive environment of the oral cavity [10]. Aside from leukotoxin other *A*. *actinomycetemcomitans* virulence genes produce fimbria, adhesins and a biofilm that provide *A*. *actinomycetemcomitans* with the ability to attach to both hard and soft tissues in the oral cavity and thus resist the forces of mastication and swift currents of saliva [17].

This *in vitro* study reports the unanticipated reduction in the expression of genes related to hard tissue binding and biofilm formation in an *A*. *actinomycetemcomitans ltx*A mutant. This decreased expression helps explain the reduced colonization of this strain in our *in vivo* model. These findings demonstrate the far-reaching effects of the mutation of one virulence gene on other apparently unrelated genes. Specifically, our results suggest that genes related to leukotoxin expression are in some way associated with genes related to fimbrial expression. Broadly these results suggest that unanticipated gene-gene interactions can be disclosed and should be examined in complex *in vivo* environments.

## Methods

### Ethics statement

BECs were collected from normal healthy human subjects approved by Institutional Review Board (IRB) of Newark Health Sciences (IRB #pro0120050257). A written consent was obtained from the subjects to participate in this study. The use of non-human primates in this study was approved by the Institutional Animal Care and Use Committees (IACUC) at Rutgers and the New England Primate Research Center (NEPRC) (IACUC#04874). Monkeys were anesthetized with 10–20 mg/kg of ketamine and medetomidine intramuscularly before sampling.

### Bacterial strains, growth conditions and plasmids

The strains and plasmids used in this study are listed in Table 1. *A*. *actinomycetemcomitans* RhAa3, a rough fimbriated, serotype ‘a’ strain was isolated from the oral cavity of a Rh monkey following the procedure described previously [10]. *A*. *actinomycetemcomitans* strains were routinely grown on Brain Heart Infusion (BHI) agar with 0.6% yeast extract (Beckton Dickinson, Franklin Lakes, NJ) supplemented with 0.8% dextrose and 0.4% sodium bicarbonate. For liquid cultures, BHI agar was replaced with BHI broth (Beckton Dickinson, Franklin Lakes, NJ). To maintain resistance, the growth media was supplemented with 50 μg/ml spectinomycin or 2 μg/ml chloramphenicol. The strains were incubated at 37°C in a 10% CO_2_ incubator for 16–48 h. *Escherichia coli* strains were routinely grown on LB media supplemented with appropriate antibiotics.

**Table 1:** Plasmids used in this study.

**Strains/Plasmids**	**Relevant genotype or characteristics**	**Source**
***Aggregatibacter actinomycetemcomitans***		
RhAa3	Wild type, serotype a	Isolated from rhesus monkey oral cavity, Labstock [10]
RhAa-VS2	RhAa Δ*ltx*A	This study
RhAa-VS3	RhAa-VS2 (pSK248)	This study
***Escherichia coli***		
Mach-1-T1^R^	*lacZΔM15 hsdR lacX74 recA endA tonA*	Invitrogen
**Plasmids**		
pBluescript KS II (+)	Amp^R^ cloning vector	Agilent Technologies
pJAK12 blue	Sp^R^, cloning vector	[42]
pSK248	Cm^R^, *ltx*A gene in pJAK16	[21]
pUZ8002	IncP Tra^-^ (*oriT1*) Km^R^ Tc^R^	[43]
pVS22	*ltxA* gene cloned in to pBluescript KS II (+)	This study
pVS24	*ltxA* insertionally inactivated with *aad*A	This study

### DNA procedures

DNA manipulations were carried out as described previously [18]. Transformation of One Shot Mach-1 T1 *E*. *coli* (Cat#C8620-03) was carried out as described in the manufacturer’s manual (Life technologies, Grand Island, NY). Transformation into *A*. *actinomycetemcomitans* was done by electroporation. *E*. *coli* transformants containing recombinant plasmids were selected on LB agar plates supplemented with the appropriate antibiotics. Plasmid DNA was isolated using the QIAprep Spin Miniprep Kit (Qiagen). Genomic DNA was isolated using the DNeasy Blood & Tissue Kit (Qiagen). Gel extractions were performed using the QIAquick gel extraction Kit (Qiagen). Restriction digestion reactions were carried out as recommended by the manufacturer (New England Biolabs). All PCR products were amplified with Phusion DNA polymerase (Thermoscientific) as recommended by the manufacturer. All oligonucleotides used in this study were synthesized from Integrated DNA technologies. The restriction enzyme sites included in the primers are underlined in the sequences (S1 Table). All primers for cloning and qPCR were designed using an online program (Oligoanalyzer 3.1, Integrated DNA technologies https://www.idtdna.com/calc/analyzer) based on the genome sequence of *A*. *actinomycetemcomitans* RHAA1 [19]. All plasmid constructs were verified by DNA sequencing (Macrogen Inc, New York NY).

### Construction of *ltx*A knockout plasmid and development of a LtxA deficient strain

Knockout of *ltx*A was carried out by amplifying a 3.6-kbp fragment comprising the *ltx*A locus derived from RhAa3 genomic DNA using *Kpn*I*ltx*F and *Sac*I*ltx*R primers (S1 Table). The amplified fragment was restriction digested with *Kpn*I and *Sac*I. The restriction digested PCR product was ligated into *Kpn*I and *Sac*I double digested pBluescript II KS (+) (Agilent technologies). The ligation mixture was transformed into One Shot Mach-1 T1 *E*. *coli*. Transformants were selected on LB agar supplemented with 100 μg/ml ampicillin, 20 μg/ml X-gal and 40 μg /ml IPTG. Approximately 10 white colonies were picked and screened for the insert by PCR and confirmed by sequencing. The resultant recombinant plasmid was designated pVS22. To disrupt the *ltx*A gene, a 6.4 kbp fragment consisting of a *ltx*A insert in conjunction with the entire pBluescript II KS (+) was amplified with *Xho*I*ltx*AF and *Not*I*ltx*R primers using pVS22 as template (Table 1). This amplification introduced the *Xho*I and *Not*I restriction sites in the *ltx*A gene to favor the introduction of the *aad*A gene. Next, a 1948 bp fragment encoding the spectinomycin resistant *aad*A gene from pJAK12 blue was amplified using *NotIaad*AF and *Xho*I*aad*AR. Both these fragments were gel purified, restriction digested with *Not*I-*Xho*I, and ligated and transformed into One Shot Mach-1 T1 *E*. *coli*. The spectinomycin resistant colonies were screened and the resultant plasmid was designated as pVS24. The plasmid pVS24 was transformed into RhAa3 by electroporation with modifications [20]. Briefly, RhAa3 was grown overnight on BHI agar plates and the cells were scraped and washed three times with ice-cold electroporation buffer (300 mM sucrose in 2.43 mM phosphate buffer, pH 7.2). The cells were then dispersed with a hand held motorized mortar and pestle (Kimble chase) and re-suspended to an OD_550_ = 0.5–0.6 in electroporation buffer. Then 40μl of cell suspension was incubated with 0.5 to 1.0 μg of plasmid pVS24 held on ice for 5 minutes and transferred to a 0.2 cm cuvette (Bio-Rad). Electroporation was carried out with Gene-pulsar (Bio-Rad) by delivering setting of 2.2 kV, 200 Ω, and 25 μF. The electroporated bacteria were immediately transferred into 1ml of warm outgrowth BHI broth media and incubated at 37°C in a 10% CO_2_ incubator for 5h. Colonies were selected on media containing 20 μg/ml Spectinomycin. Further, the double cross over event of pVS24 was confirmed by PCR. The resultant *ltx*A knockout strain was designated as RhAa-VS2.

### Genetic complementation of *ltx*A

For complementation, plasmid pSK248, a derivative of pJAK16 containing *ltx*A ORF was used [21]. This plasmid was mobilized into *A*. *actinomycetemcomitans* as previously described [22]. The resultant transconjugant was designated as RhAa-VS3. Expression of *ltx*A was carried out in BHI broth containing 0.5 mM IPTG in an anaerobic chamber. Since RhAa3 is a minimal LtxA producer during growth in a10% CO_2_ incubator, the strains were incubated in anaerobic chamber for leukotoxin detection by THP-1 cell killing assay.

### THP-1 cell killing assay

THP-1 cells obtained from ATCC were used for this assay. The cells were grown in RPMI media supplemented with 10% fetal bovine serum and incubated in a 5% CO_2_ incubator. The cell-killing assay was done as described previously [23]. Briefly, 5×10^6^ cells/ml were used for the assay. *A*. *actinomycetemcomitans* strains RhAa3, RhAa-VS2 and RhAa-VS3 were grown in a 10% CO_2_ incubator or anaerobically in BHI broth. Extracellular culture supernatant and the whole cell lysate were collected as the source of secreted and un-secreted leukotoxin and filter-sterilized using 0.2μm filters. To determine the cell killing effect of leukotoxin produced by *A*. *actinomycetemcomitans* strains, the culture supernatant or the whole cell lysate were mixed with 500 μl of THP-1 cells (~10^6^ cells/ml) and the mixture was incubated at 37°C, 5% CO_2_ for 3 hours. Purified leukotoxin and BHI broth with supplements were used as positive and negative controls respectively. Cellular viability (ATP production) was then determined using the CellTiter-Glo luminescent cell viability assay (Promega, Madison, WI) according to the manufacturer’s instructions. Plates were read in a Tecan infinite 200 PRO plate reader in the luminescence mode (Tecan Austria GmbH, Austria). Cytotoxicity assays were performed at least three different times. Significant difference in cell killing assay was calculated by one-way ANOVA with Tukey’s post-hoc multiple comparison test using Graph pad prism 6.0. A *P*<0.05 was considered as significance.

### Hydroxyapatite (HA) beads binding Assay

The HA binding assay was performed as previously described [10]. Briefly, *A*. *actinomycetemcomitans* strains were grown on BHI agar supplemented with appropriate antibiotics and re-suspended in PBS to achieve an optical density A_560_ = 0.9 (equivalent to 1×10^8^ cells/ml). Whole unstimulated saliva was collected from normal healthy subjects approved by Institutional Review Board (IRB) of Newark Health Sciences. The saliva was clarified by centrifugation at 10,000 × g for 10 min to obtain saliva for addition to 50 mg HA beads (BDH Chemicals Ltd. Poole, UK). A written consent was obtained from the subjects to participate in this study. The beads were first washed three times with PBS. The 50mg of washed HA beads were coated with 500 μl of clarified saliva by incubating the beads with saliva for 20 minutes on a rotating device (RotoTorque from Cole Palmer Instruments, Chicago IL). The saliva coated HA (SHA) beads were then washed twice with 500 μl of PBS and the beads were air-dried. 300 μl of a bacterial suspension (including strains; RhAa3, RhAa-VS2 and RhAa-VS3) was treated with 50mg of SHA beads and the mixture was incubated for 1h on the rotating device. After 1h incubation, the mixture was centrifuged at 300-X g for 2 minutes. The supernatant containing the unbound bacteria was serially diluted and plated. The pellet was washed with PBS twice to remove all unbound bacteria. After the final wash, the bacterial pellet was suspended in PBS and sonicated for 30 cycles (20% amplitude, 0.5 second) followed by centrifugation at 300-X g for 2 minutes. The supernatant containing the previously SHA bound bacteria was serially diluted and plated. The statistical significance for the ratio of bound to unbound *A*. *actinomycetemcomitans* was calculated by one-way ANOVA with Tukey’s post-hoc multiple comparison test. A *P*<0.05 was considered as significance.

### Total *A*. *actinomycetemcomitans* biofilm RNA extraction and purification

RNA isolation from *A*. *actinomycetemcomitans* strains was carried out as described previously [24]. The RNA samples were purified by passing the samples through Micro Bio-Spin P-30 Gel Columns (Bio-Rad). RNA quantification was done using the Nanodrop Lite spectrophotometer (Thermofisher Scientific, Wilmington, DE) and the integrity was assessed on 1% agarose gel. Further, genomic DNA contamination was removed by treating the RNA samples with DNaseI and a RNA purification Kit (Zymo Research, Irvine). Finally, removal of genomic DNA contamination was confirmed for every RNA extraction by PCR with 5SrRNA specific primers before proceeding to cDNA synthesis. A reaction with genomic DNA was used as the positive control.

### Quantitative PCR

Synthesis of cDNA from total RNA was performed using the High capacity reverse transcription kit (Applied biosystems) according to the manufacturer’s instructions. A 25 μl qPCR reaction using the cDNA template was performed using Roche SYBR green master mix in LightCycler 480 system as described in the user manual. Primers used for the qPCR are listed in S1 Table. 5s rRNA was used as the normalization control. Melting curve analysis was done to analyze the specificity of the amplified product. Data analysis was done using LightCycler 480 software (Version 1.2.9.11). A reaction without reverse transcriptase was always performed as a negative control. Results were described by reporting the standard errors of the means (±SEM) calculated from triplicate experiments. Data was analyzed by one-way ANOVA with Tukey’s post-hoc multiple comparison test. A *P*<0.05 was considered as significance.

### Congo red staining and assessment of exo-polysaccharide (EPS) production

*A*. *actinomycetemcomitans* strains were grown in a 12-well plate in BHI broth with supplements for 16 h. The supernatant was discarded and the biofilm was stained with 2mg/ml of congo red solution in water for 20 min. Excess stain was removed by rinsing the wells with distilled water and the intensity of the congo red staining was photographed using Olympus SZ61 microscope at 4.5× magnification. After drying the plates, the wells were de-stained with water and absorbance of the suspension was read in a microplate reader (Tecan) at OD_415 nm_ [25]. Experiments were conducted in triplicate and the significant difference in EPS production between the strains was calculated by one-way ANOVA with Tukey’s post-hoc multiple comparison test. A *P*<0.05 was considered as significance.

### Confocal Microscopic analysis of biofilm depth and live/dead staining

Confocal microscopy was done as described previously [26]. Briefly, *A*. *actinomycetemcomitans* strains were inoculated in 35mm glass bottom micro well culture dishes (Cat. # P35G-0-10-C.s, MatTek Co. Ashland, MA) and grown for 16 h and 48 h in BHI broth. The biofilm was washed with fresh media followed by staining with Film tracer biofilm LIVE/DEAD stain (Life Technologies, NY). The biofilm was incubated with the stain for 20 min in the dark. After washing the cells with media, confocal imaging was done using a Nikon A1R-A1 confocal microscope and an objective lens Plan Apo VC 60X WI DIC N2. For 16 h biofilm the image (20X) was used to analyze biofilm depth at 9–11 different zone. The 48 h biofilm image (60X) was used for live/dead cells. In addition, the 16 h and 48 h biofilms were serially diluted; plated and colony-forming units were enumerated to validate the live/dead confocal imaging data. All data were analyzed by one-way ANOVA with Tukey’s post-hoc multiple comparison test. A *P*<0.05 was considered as significance.

## Results

### Characterization of *ltx*A knockout strain (RhAa-VS2) isolated from Rhesus monkey

**Fig 1:**
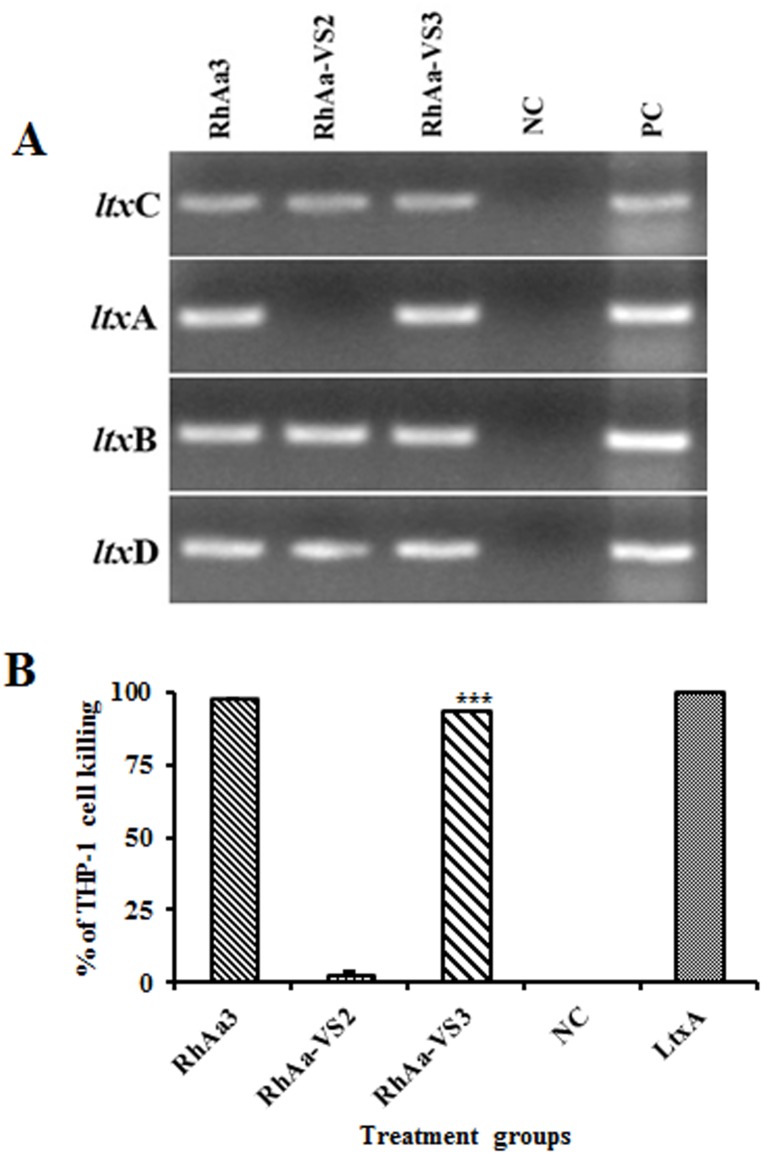
RT-PCR of *ltx* operon genes in *A*. *actinomycetemcomitans* strains and leukotoxic activity by THP-1 cell killing. The RT-PCR analysis shows that there was no *ltx*A transcript in RhAa-VS2, however the other genes in the *ltx* operon were intact without polar effect. The complemented strain RhAa-VS3 showed an *ltx*A band corresponding to RhAa3. NC- negative control reaction with RNA as the template without reverse transcriptase. PC-positive control with DNA as the template (A). The extracellular extracts of the anaerobically grown RhAa-VS2 strain showed a significantly decreased leukotoxicity (*P*<0.0001) against human macrophage cell line THP-1 when compared to RhAa3 strain. The complemented strain RhAa-VS3 (*P*<0.0001) strain restored leukotoxic activity, which was confirmed by cell killing assay. NC- BHI broth was used as the negative control. LtxA-purified leukotoxin used as the positive control. Results were analyzed by one-way ANOVA with Tukey’s post-hoc multiple comparison and (*) *P*<0.05 was considered as significance (B).

The *ltx*A knockout plasmid (pVS24) was constructed by insertional inactivation of the cloned *ltx*A gene of RhAa3 using a PCR amplified spectinomycin (*aad*A) cassette. Upon transformation of pVS24 into RhAa3, the *ltx*A chromosomal locus was replaced with *aad*A gene. To confirm that the *ltx*A knockout did not have polar effects on the other genes in the ltx operon, we carried out RT-PCR for *ltx* operon genes such as *ltx*C, *ltx*B and *ltx*D. The RT-PCR results showed that there was no *ltx*A expression in RhAa-VS2 strain. Further, the knock out of *ltx*A gene did not affect the expression levels of *ltx*C, *ltx*B and *ltx*D in RhAa-VS2 compared to RhAa3 strain. The *ltx*A complemented strain showed the *ltx*A amplicon corresponding to RhAa3 demonstrated by RT-PCR (Fig 1A).

### THP-1 macrophage killing activity

Since the wild type RhAa3 strain is a minimal LtxA producer, we used a sensitive THP-1 macrophage cell-killing assay to confirm the knockout of *ltx*A gene [23]. The results showed that LtxA production was significantly decreased in the extracellular fraction of RhAa-VS2 strain (*P*<0.0001) when compared to RhAa3 strain (Fig 1B). Further intracellular fractions also confirmed that there was no leukotoxin produced by RhAa-VS2 strain (Data not shown). The leukotoxic activity was restored upon genetic complementation of *ltx*A in RhAa-VS3 (*P*<0.0001) when compared to RhAa-VS2 (Fig 1B).

### Hard tissue binding abilities

**Fig 2:**
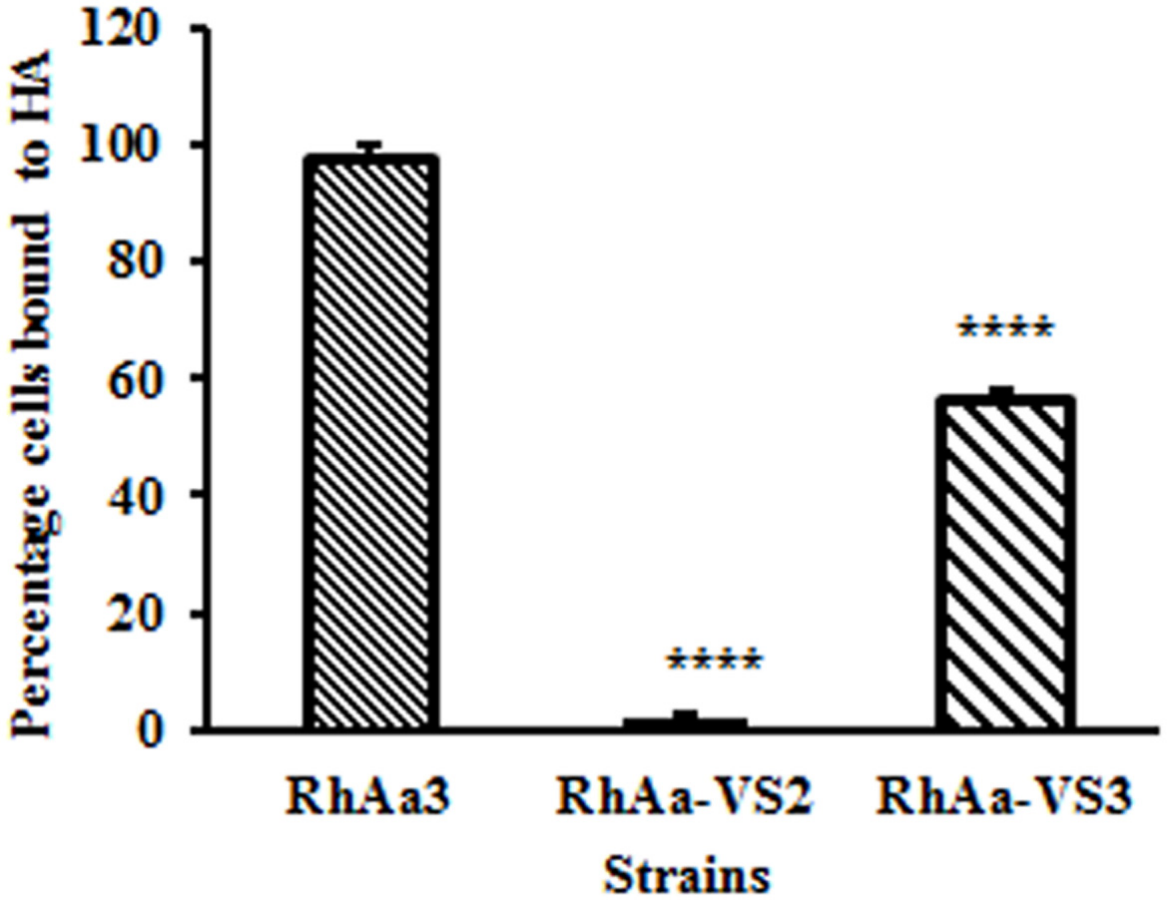
Hydroxyapatite binding assay. The hard tissue binding comparing the ratios of bound to unbound *A*. *actinomycetemcomitans* strains to HA showed a significant difference in their binding efficiencies. The RhAa-VS2 strain showed a significantly decreased binding ability compared to RhAa3 (Fig, *P*<0.006). The complemented strain RhAa-VS3 strain showed a significant increase (*P*<0.002) in binding compared to RhAa-VS2. Significant differences in binding abilities of the strains were calculated by one-way ANOVA with Tukey’s post-hoc multiple comparison test. *P<0.05 was considered as significance.

Binding assays were carried out with RhAa-VS2 and RhAa3 strains in order to determine the effect of the *ltxA* knock-out on binding to BECs and SHA. The BEC binding assay revealed that there was no significant difference in the binding efficiencies between RhAa3 and RhAa-VS2 strains (data not shown). However, the SHA binding assay showed that there was a significantly decreased (*P =* 0.006) binding of RhAa-VS2 strain to SHA when compared to the RhAa3 strain (Fig 2). The *ltx*A complemented strain showed a significant increase (*P =* 0.002) in SHA binding when the RhAa-VS3 strain compared to RhAa-VS2.

### Expression levels of genes related to salivary hydroxyapatite attachment

**Fig 3:**
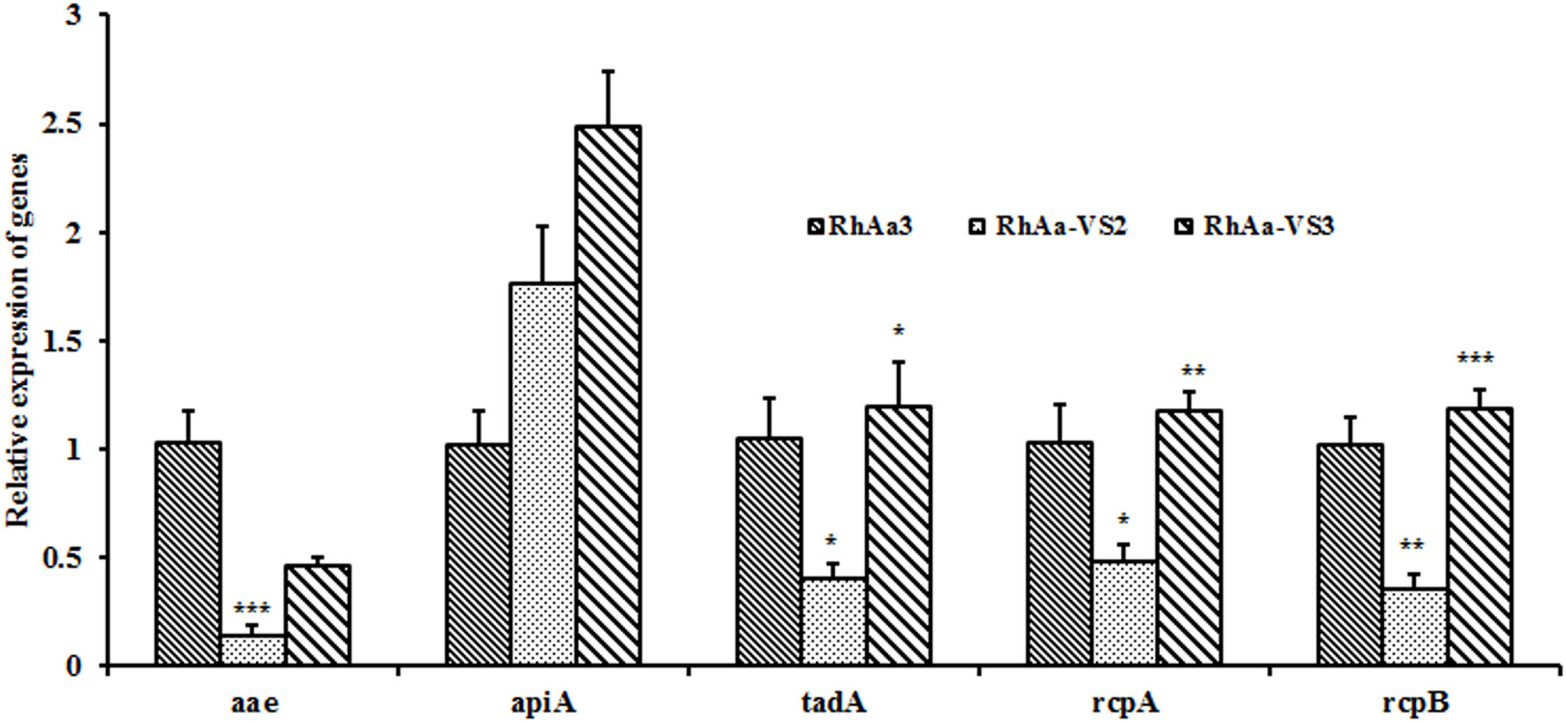
qRT-PCR analysis of genes related to salivary hydroxyapatite attachment. There was a significant decrease in the expression of *aae* (*P* = 0.0002) and tight adherence operon genes such as *tad*A (*P* = 0.0468), *rcp*A (*P* = 0.0184) and *rcp*B (*P* = 0.002) in RhAa-VS2 strain when compared to wild type RhAa3. The expression level of *api*A was not affected in RhAa-VS2 compared to RhAa3. The expression levels of these genes were also compared between RhAa3, RhAa-VS2 and RhAa-VS3. One-way ANOVA analysis of the results showed that the genes in the *tad*A locus were significantly increased (*rcp*A, *P* = 0.005; *rcp*B, *P* = 0.0004; *tad*A, *P* = 0.0166) in RhAa-VS3 compared to RhAa3 and RhAa-VS2. However, the levels of *aae* and *api*A did not significantly increase up on complementation. Significant differences in gene expression levels were calculated by one-way ANOVA with Tukey’s post-hoc multiple comparison test. *P<0.05 was considered as significance.

To understand the biological rationale for the decreased binding of the strain RhAa-VS2 we examined the mRNA expression of tight adherence (*tad*) and adhesin genes. To do so we used real time PCR to examine the expression of genes from 16 h biofilms. There was a significant decrease in the expression of *aae* (*P* = 0.0002) and tight adherence genes such as *tad*A (*P* = 0.0468), *rcp*A (*P* = 0.0184) and *rcp*B (*P* = 0.002) in RhAa-VS2 strain when compared to wild type RhAa3. The expression level of *api*A was not affected in RhAa-VS2 compared to RhAa3. The expression levels of these genes were also compared between RhAa3, RhAa-VS2 and RhAa-VS3. One-way ANOVA analysis of the results showed that the genes in the *tad*A locus were significantly increased (*rcp*A, *P* = 0.005; *rcp*B, *P* = 0.0004; *tad*A, *P* = 0.0166) in RhAa-VS3 compared to RhAa3 and RhAa-VS2 (Fig 3). Significant differences in gene expression levels were calculated by one-way ANOVA with Tukey’s post-hoc multiple comparison test. *P<0.05 was considered as significance.

### Biofilm depth and congo red staining

**Fig 4:**
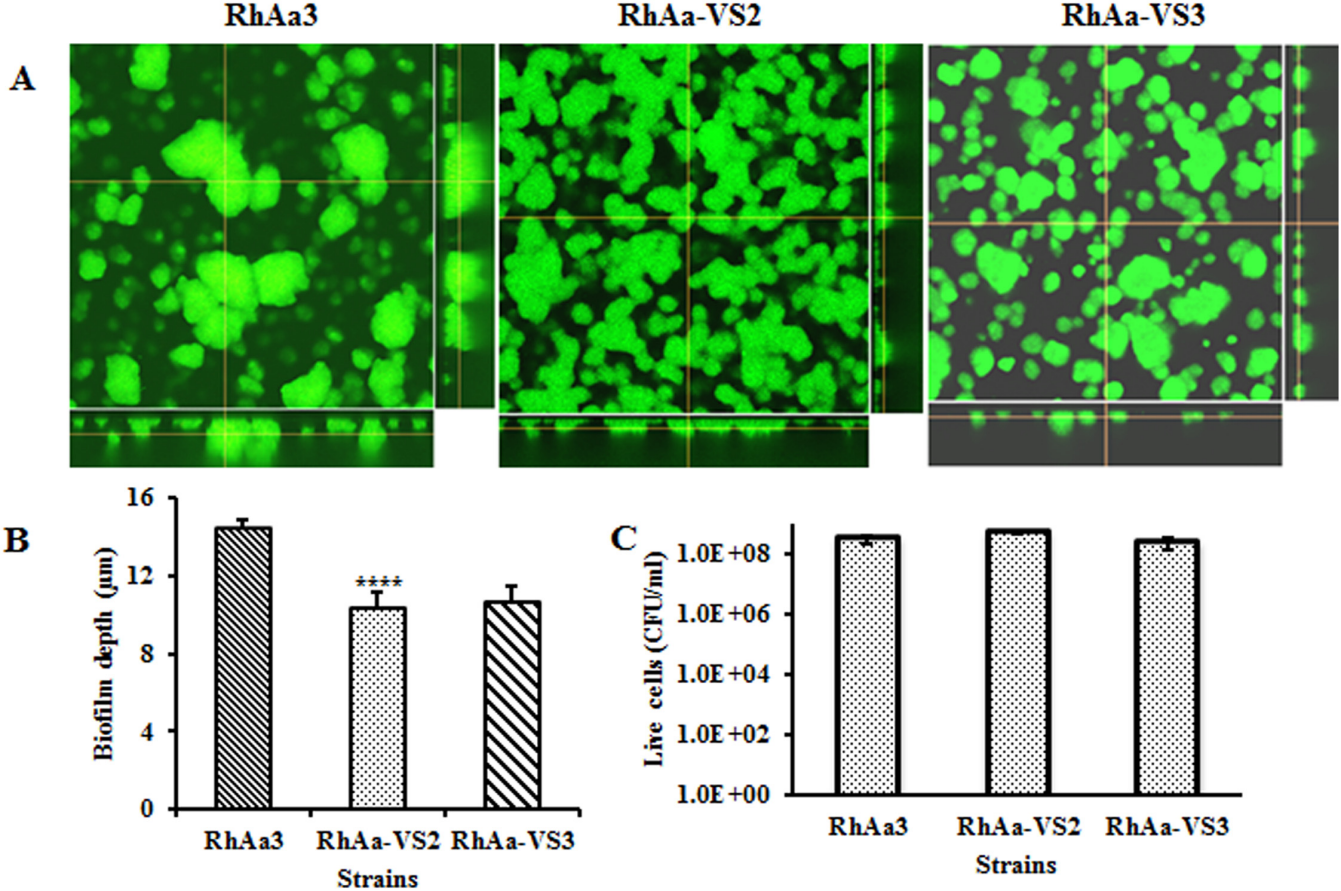
Confocal microscopic measurement of biofilm depth. Biofilm depth was determined by performing Z- axis plane scans and determined by integrating fluorescence intensity across the Z-stack image (A). The biofilm depth was significantly reduced (*P* = 0.008) in RhAa-VS2 and RhAa-VS3 strains compared to RhAa3. The complemented strain RhAa-VS3 showed no significant increase in biofilm depth (B). The 16 biofilm cells were serially diluted and plated. Data shows CFU from different strains (C). Significant differences in biofilm depths and CFU were calculated by one-way ANOVA with Tukey’s post-hoc multiple comparison test. *P<0.05 was considered as significance.

Biofilms of RhaAa-VS2 and RhAa3 strains were grown for 16 h and compared using confocal microscopy. The images showed that both the strains exhibited similar typical 3D biofilm structure (Fig 4A). However, there was a significant decrease (*P =* 0.008) in biofilm depth and CFU/ml observed in RhAa-VS2 strain as compared to wild type strain. There was no significant increase in biofilm depth in complemented strain RhAa-VS3 (Fig 4B). In order to analyze whether the biofilm depth associated with CFU, we serially diluted the 16 h biofilm cells and results showed that there was no significant difference between the RhAa3, RhAa-VS2 and RhAa-VS3 (Fig 4C).

**Fig 5:**
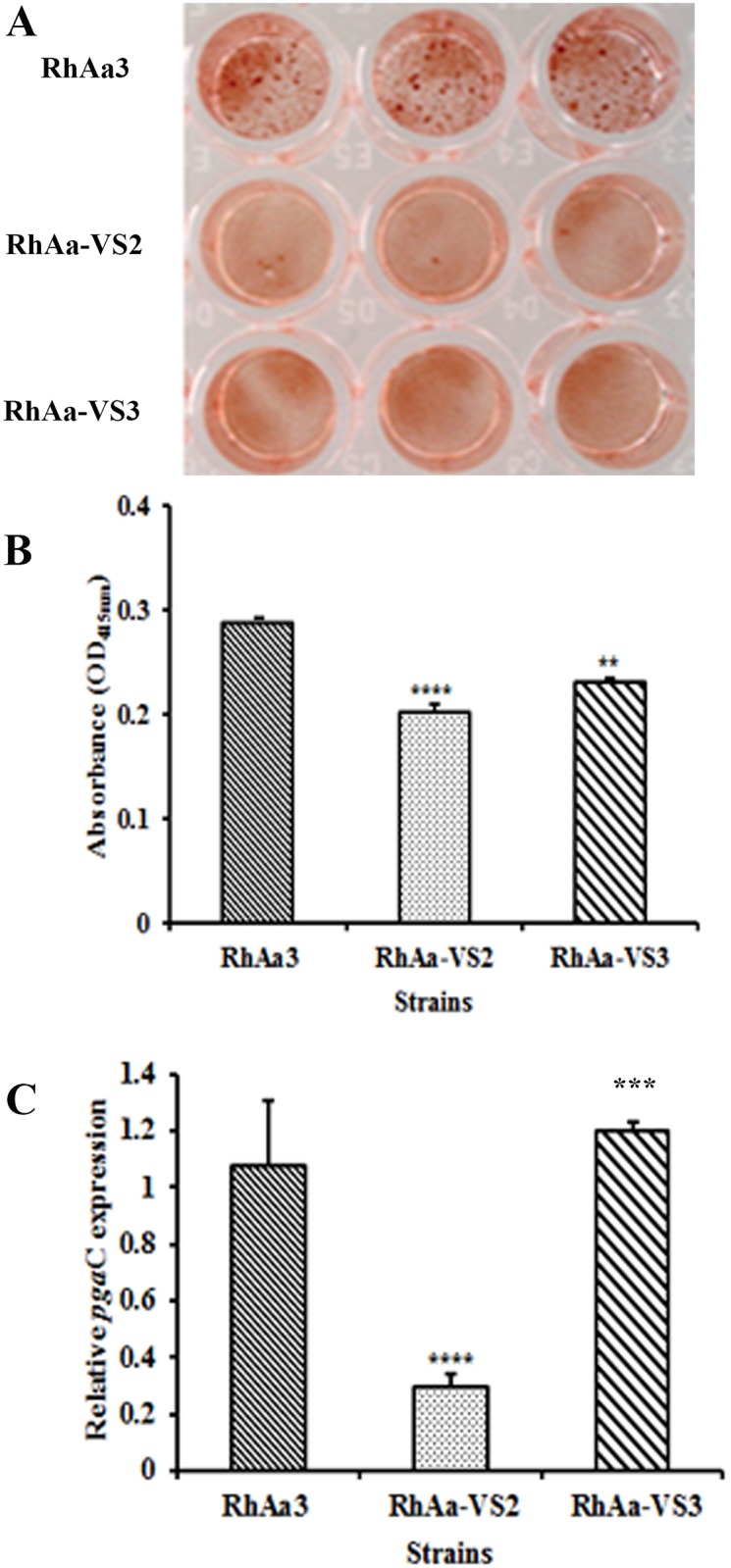
Biofilm congo red staining and expression of *pga*C gene. Staining of the biofilm using congo red image (A) show that there was a decreased EPS production in RhAa-VS2. Data of bound congo red on to the biofilm (B) show that RhAa-VS2 strain produced significantly lower (*P*<0.0001) level of EPS production compared to RhAa3. Further, the EPS production was restored in RhAa-VS3 strain (*P* = 0.0035). The result of qRT-PCR (C) show that the genes responsible for EPS production, *pga*C was significantly decreased in RhAa-VS2 (*P* = 0.0076) compared to RhAa3, which was restored in RhAa-VS3 (*P* = 0.0031) using 5srRNA gene as the internal control.

In addition we also analyzed one of the major biofilm components, EPS production, since the RhAa-VS2 strain exhibited decreased biofilm formation compared to RhAa3. The 16 h grown biofilm was stained with congo red to measure the bound dye (Fig 5A), which is proportional to the amount of EPS production [27]. The RhAa-VS2 strain produced a significantly low level (*P*<0.0001) of EPS as compared to RhAa3. This was restored in complemented strain RhAa-VS3 (*P* = 0.0035) (Fig 5B). Furthermore, qRT-PCR results also confirmed that the expression of the gene responsible for exopolysaccharide production (*pgaC)* was significantly decreased in the RhAa-VS2 strain (*P* = 0.0076) when compared to RhAa3 and the expression was restored in RhAa-VS3 compared to RhAa-VS2 (*P* = 00031) (Fig 5C).

### Viability of biofilm cells

**Fig 6:**
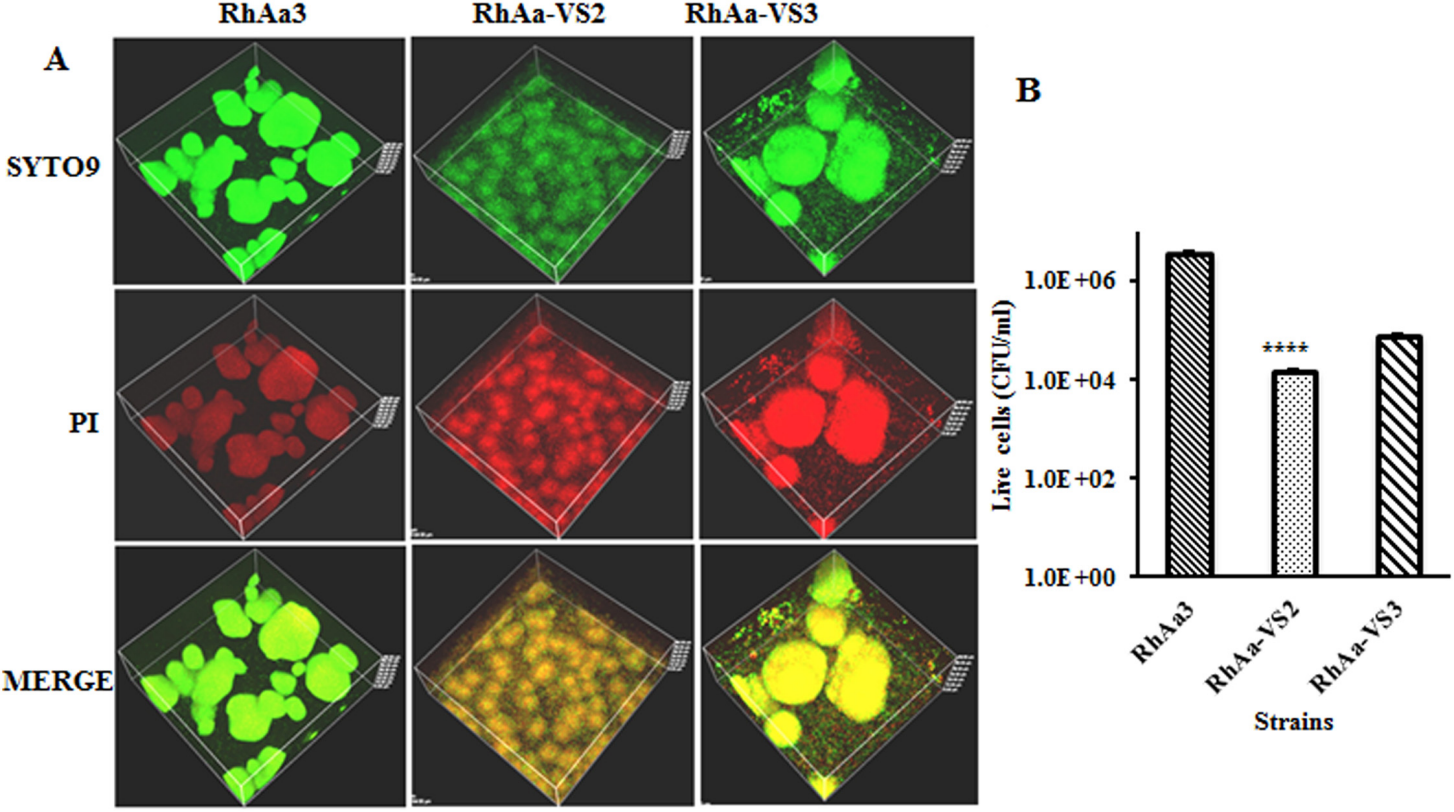
Live/dead biofilm cells at 48 h growth. Film tracer Live/dead stained confocal images (A) show that there was more number of dead cells in RhAa-VS2 (*P*<0.0001) compared to RhAa3. The RhAa-VS3 strain did not show any difference in cell viability at 48 h compared to RhAa-VS2 strain. The plate count colony forming units at 48 h of biofilms also show that there was a significant decrease (*P*<0.0001) in the number of RhAa-VS2 viable cells compared to RhAa3. The cell viability of RhAa-VS3 (Complemented strain) did not show any significant difference compared to RhAa-VS2 strain (B).

The confocal images of the 16 h (Data not shown) biofilm cells were showed that there were no significant differences between RhAa3, RhAa-VS2 and RhAa-VS3 strains (Fig 4C). However, analysis of the images of 48 h biofilm showed that there was a significantly decreased viable cell in RhAa-VS2 strain compared to RhAa3 (*P*<0.0001, 98.9±0.47%live; 1.13±0.47% dead cells in RhAa3; 69.2±0.16% live; 30.8±0.16% dead cells in RhAa-VS2) (Fig 6A). The RhAa-VS3 strain did not show any difference in cell viability at 48 h compared to RhAa-VS2 strain (66.96±2.78% live; 33.03±2.78% dead cells in RhAa-VS3). The plate count colony forming units at 48 h of biofilms also show that there was a significant decrease (*P*<0.0001) in the number of RhAa-VS2 viable cells compared to RhAa3. The cell viability of RhAa-VS3 strain did not show any significant difference compared to RhAa-VS2 strain as measured by CFU enumeration (Fig 6B).

## Discussion

Leukotoxin is a key virulence factor produced by *A*. *actinomycetemcomitans* and linked to the aggressive nature of bone loss seen in subjects with localized aggressive periodontitis [28]. The most well characterized phenotypic trait attributed to leukotoxin is its ability to kill PMNs, lymphocytes and macrophages [29]. Initial recognition of the importance of Ltx was derived from *in vitro* studies[30]. These results were re-inforced by clinical data describing the potential for increased risk for aggressive periodontitis in Moroccan adolescent subjects who harbored the higher leukotoxin producing JP2 strain of *A*. *actinomycetemcomitans* as compared to those who had the minimally toxic *A*. *actinomycetemcomitans* strain[31].

Our group developed a Rh monkey model designed to examine *A*. *actinomycetemcomitans* colonization and survival in a competitive oral environment. Our overarching goal was to; 1) show how we could inoculate a labeled strain of *A*. *actinomycetemcomitans* into the oral cavity and recover it over time, and 2) interrupt key *A*. *actinomycetemcomitans* virulence genes and assess their effect on colonization and survival of that strain over time. As such we interrupted structural genes in *A*. *actinomycetemcomitans* with the targeted insertion of a spectinomycin resistance cassette and then assessed the effect of this alteration on survival in an oral environment known to support the growth and survival of *A*. *actinomycetemcomitans*. A Rh monkey strain (RhAa-VS2) with a leukotoxin (*ltxA*) mutation failed to colonize the Rh oral cavity while a RhAa strain with a *luxS* mutation (deficient in a quorum sensing) and its wild type parental strain (RhAa3) did colonize and survive (Unpublished data). This finding was unexpected and led us to test the effect of a *ltx* knockout on *A*. *actinomycetemcomitans* binding and biofilm formation in a well controlled *in vitro* setting.

Attachment of *A*. *actinomycetemcomitans* to hard and soft tissues is mediated by separate operons, the *tad* operon (contained within the 14 gene operon known as the widespread colonization island), which controls binding to teeth and hard tissues [32] and autotransporter proteins, Aae [9] and ApiA [33], which are the dominant protein adhesins that mediate attachment to soft tissues such as BECs [34]. In addition, biofilm integrity is determined by extracellular polysaccharide mediated by *pagC* contained within the *pag* operon (26). Our *in vitro* results showed that binding to salivary coated (SHA) by the *ltx* mutant strain (RhAa-VS2) was significantly reduced. mRNA qRT-PCR results indicated that all *A*. *actinomycetemcomitans* genes we tested that were related to SHA binding were downregulated in RhAa-VS2 strain. From these results we concluded that binding of RhAa-VS2 strain to SHA beads was likely due to the cumulative downregulation of *tad* genes in the widespread colonization island of *A*. *actinomycetemcomitans* [35]. Moreover, complementation restored these attachment capabilities. In contrast, genes responsible for soft tissue attachment were minimally effected. While biofilm formation was downregulated in RhAa-VS2 strain and it was not fully restored in the complemented strain.

Previous results from a rat model also demonstrated that the *A*. *actinomycetemcomitans* strains mutated *tad*A (or *flp-1* also part of the widespread colonization island) failed to colonize the rat’s oral cavity [36]. Further, *in vitro* studies have demonstrated that an *A*. *actinomycetemcomitans flp*-1 mutant strain retains it’s binding to BECs but completely looses its ability to bind to SHA [9]. Our *in vitro* results also indicate that unlike hard tissue binding, soft tissue or BEC binding was not altered in RhAa-VS2 strain (data not shown). It seems reasonable to speculate that BEC binding, which was unchanged in RhAa-VS2 mutant, may not have been necessary for survival of *A*. *actinomycetemcomitans* in the oral cavity of monkeys since these genes were unaffected in RhAa-VS2 strain. Furthermore, *A*. *actinomycetemcomitans* strains producing these adhesins still failed to colonize or survive. These results agree with findings in the rat colonization model and in human and *in vitro* experiments where *tad* related binding to teeth appeared to be more tenacious and critical than adhesins related binding to BECs [9].

To exclude the possibility of polar effects due to the *ltx*A insertional inactivation and/or mutation, we performed RT-PCR and qRT-PCR analysis for the all other genes in the *ltx* operon. The results showed that there was no polar effect as a result of the *aad*A insertion (SpecR) in the *ltx* operon, which was done to label *A*. *actinomycetemcomitans in vivo*. We were also concerned with the effect that the *ltx*A mutation might have on cell viability and growth. Our results showed that no cell death occurred at 16 h of growth but cell death was seen at 48 h of growth in RhAa-VS2. As a result all assessments for RhAa-VS2 strain and RhAa3 cells were performed at the 16 h time point. Confocal microscopy and CFU analysis supported this conclusion in that there was no difference in the viability of the cells after 16 h; however, 48 h biofilm cells did reveal substantial cell death (26).

Biofilms appear to be important in *in vivo* studies of adherence [37]. Biofilms are adherent bacterial communities that display a distinctive physiological state often protecting bacteria from hostile environmental conditions [37, 38]. In the current study, confocal microscopy revealed that the RhAa-VS2 strain exhibited less biofilm depth and a different architecture. Typically cells found within a biofilm matrix are encased in a protective extracellular polymeric substance (EPS; [39, 40]). In the case of RhAa-VS2, the mRNA expression level of *pgaC*, the gene responsible for EPS, which is consistent with the 50% reduction seen in EPS in this strain.

Therefore, two components necessary for biofilm formation, *tadA* and *pga*C were both downregulated in the RhAa-VS2 strain. Nevertheless, only the *tad* genes were restored after complementation, however biofilm mass was not fully restored. It has been shown that *A*. *actinomycetemcomitans* extracellular polysaccharide is not essential for the adhesion of cells to surfaces but functions primarily in the intercellular aggregation process [41]. These results once again support the concept that the mutated *ltx*A gene had a direct effect on genes related to SHA binding and also a profound effect on biofilm depth restoration.

To summarize at this juncture, we have powerful evidence that a knock-out in the leukotoxin structural gene, *ltxA*, has an effect on attachment related virulence genes in *A*. *actinomycetemcomitans*. These experiments provide evidence of the value derived from animal experiments that can illustrate the unexpected reach of specifically targeted genes. Most importantly this data provides a rationale for using both *in vitro* and *in vivo* data to clarify the effect that mutation in one virulence gene has on other apparently unrelated genes. This approach may help us to understand gene expression in an ecologically competitive environment that can influence the balance between health and disease. We are currently using global transcriptomic analysis and RNA seq in an effort to examine genes that are up and downregulated by comparing the RhAa-VS2 strain to its wild type parental counterpart.

## Conclusion

Based on the results to date we can conclude that the RhAa-VS2 strain;

1. Reduces leukotoxin production by interfering with transcription, which we determined was independent of any polar or cell viability effects.
2. Reduces the ability to bind to hard tissue but has little to no effect on the strains ability to bind to soft tissue.
3. Reduces biofilm forming capability.

Since complementation restores the production of leukotoxin and the ability of the altered strain to bind to SHA and to restore the biofilm formation., We conclude that this leukotoxin mutation had a direct effect on genes related to *A*. *actinomycetemcomitans* colonization.

Taken together, our *in vitro* findings helped explain the *in vivo* observation that the RhAa-VS2 strain failed to colonize the oral cavity in our Rh monkey colonization model [10]. Therefore, the in-depth *in vitro* exploration of the physiological reach of the *ltx*A gene interruption assessed in this study helped us explain the finding that a spectinomycin labeled insertion that created a *ltx*A deficient *A*. *actinomycetemcomitans* strain failed to colonize the mouths in our Rh animal model.

## Supporting Information

S1 Table (DOCX)
