# Supplementary material for: Profound Effects of Aggregatibacter actinomycetemcomitans Leukotoxin Mutation on Adherence Properties Are Clarified in in vitro Experiments
Source: PLoS One. 2016 Mar 15;11(3):e0151361. doi: 10.1371/journal.pone.0151361 (PMC4792451; doi:10.1371/journal.pone.0151361)
Supplement: S1 Table — (DOCX) [file pone.0151361.s001.docx]

S1 Table. Oligonucleotides used in this study

| **Oligonucleotides** | | | **Sequence (5’→3’)** | | **Tm ^o^C** |
| --- | --- | --- | --- | --- | --- |
| ***ltx*A knockout plasmid construction** | | | | | |
| *aad*A*Not*I | | TGAAAGCGGCCGCTTATTTGCCGACTACCTTGG | | 67.4 | |
| *aad*A*Xho*I | | GTGCACTCGAGAAGTGCGGTCATTCAAATATGTATCCGC | | 65.7 | |
| *Kpn*I*ltx*F | | CATCAGGTACCATGTTAGGGTATGTGGCTTGGTTATGGGC | | 66.3 | |
| *Sac*I*ltx*R | | ACTATGAGCTCATTGGTCTGCAATGGCGAAGCGGATAACG | | 67.4 | |
| *Xho*I*ltx*AF | | GTTGTCTCGAGTTATTTGCAAGTAATAGAGCCGC | | 59.1 | |
| *Not*I*ltx*AR | | AAATGGCGGCCGCTTTGTCTTTCCTAGGTATTGC | | 61.6 | |
| **Mutant screening primer** | | | | | |
| *aad*AscreenF | GCATTTGGTACAGCGCAGTAACC | | | 58.9 | |
| *aad*AscreenR | CAAGGATCTTACCGCTGTTGAGATCC | | | 59.0 | |
| *ltx*screenF | TACCCTTCTACCGTTGCCATGG | | | 58.9 | |
| *ltx*screenR | GCTGCAAGCAACCATTTAGTTTGG | | | 57.6 | |
| **qRT-PCR primers** | | | | | |
| *aae*qF | | | GGTTTTAGGCGGCACATTTA | 53.5 | |
| *aae*qR | | | TGCTTGACCAACCATAACCA | 54.4 | |
| *api*AqF | | | GCCGAGTCAATGAATTAGACAAAG | 56.4 | |
| *api*AqR | | | CAACAGCTGCACTCAAGTTAAGG | 54.2 | |
| *ltx*AqF | | | GCGGCTCTATTACTTGCA | 58.6 | |
| *ltx*AqR | | | GCAATACCTAGGAAAGAC | 47.0 | |
| *ltx*BqF | | | CCAAGATCGTACCGTGCTTA | 54.4 | |
| *ltx*BqR | | | TCTTGATGCTTGCCTTGTTC | 53.4 | |
| *ltx*CqF | | | GGGCAAATTTAAGCCTTGAG | 52.1 | |
| *ltx*CqR | | | GCGCAATCCAATCAATAAAC | 50.4 | |
| *ltx*DqF | | | GAAATTATCGCAACCGCTTC | 52.3 | |
| *ltx*DqR | | | ACGTATTCGCCCTCTTTCAC | 54.9 | |
| *pga*qF | | | GACGGTGATGCGGTATTGG | 56.1 | |
| *pga*qR | | | GACCGATGATGGAGCTGAA | 54.4 | |
| *rcp*AF | | | TGGGCATTAACTGGAGCCAC | 57.7 | |
| *rcp*AR | | | ATCCACCTCCGAAACCGAAG | 57.1 | |
| *rcp*BF | | | TCCATTGCATTGGCCTCAA | 55.1 | |
| *rcp*BR | | | CAACAGTCGTATCGTTAACAAATTCA | 53.9 | |
| *tad*AF | | | TGGCAGCCGTTTAAATGTTG | 54.2 | |
| *tad*AR | | | ATTTCGCGAGTCATAGAACC | 52.4 | |
| 5SrRNAF | | | GCGGGGATCCTGGCGGTGACCTACT | 68.0 | |
| 5SrRNAR | | | GCGATCTAGACCACCTGAAACCATACC | 60.4 | |

Sequences underlined are the restriction enzyme recognition sites
